# A survey of Asian Eye Institutions on perioperative antibiotic prophylaxis in cataract surgery

**DOI:** 10.1007/s10792-023-02816-w

**Published:** 2023-08-01

**Authors:** Prashant Garg, Wei-Boon Khor, Aravind Roy, Donald Tiang-Hwee Tan, For the APAX consortium, Yao Ke, Xiangjia Zhu, Alvin L. Young, Haripriya Aravind, Namrata Sharma, Susi Heryati, Johan A. Hutauruk, Ratna Sitompul, Kohji Nishida, Yoshinori Oie, Takefumi Yamaguchi, Khairidzan Mohd. Kamal, Irfan Jeeva, Jessica Marie R. Abaño, Ruben Lim Bon Siong, Joon Young Hyon, Kyoung Yul Seo, Shu-Wen Chang, Fung-Rong Hu, Catherine Jui-Ling Liu, Chi Chin Sun, Ngamjit Kasetsuwan, Pinnita Prabhasawat, Le Xuan Cung, Quoc Dat Nguyen

**Affiliations:** 1 LV Prasad Eye Institute, Hyderabad, India; 2 Singapore National Eye Centre, Singapore, Singapore; 3 Duke-NUS Graduate Medical School, Singapore, Singapore; 4 LV Prasad Eye Institute, Vijayawada, Andhra Pradesh India; 5 Eye and Cornea Surgeons, Eye and Retina Surgeons, Camden Medical Center, 1 Orchard Boulevard, #13-03, Singapore, 248649 Singapore

**Keywords:** Cataract, Infection, Prophylaxis, Perioperative, Asia

## Abstract

**Purpose:**

To determine current institutional practice patterns for the use of perioperative antibiotics and other measures to prevent infection after cataract surgery in Asia.

**Methods:**

An online survey-based study of leading eye institutions in China, Hong Kong, India, Indonesia, Japan, Malaysia, Pakistan, Philippines, Singapore, South Korea, Taiwan, Thailand and Vietnam was conducted. The survey was administered to 26 representative key opinion leaders from prominent tertiary eye institutions that are also national academic teaching institutions in Asia. Survey responses were collated and anonymized during analysis.

**Results:**

All surveyed institutions used povidone iodine for the preoperative antiseptic preparation of the eye, with notable variations in the concentration of povidone iodine used for conjunctival sac instillation. Preoperative topical antibiotics were prescribed by 61.5% and 69.2% of institutions in low-risk and high-risk cases, respectively. Regarding the use of intra-operative antibiotics, 60.0% and 66.7% of institutions administered intracameral antibiotics in low-risk and high-risk patients, respectively. Postoperative topical antibiotics use patterns were generally very similar in low-risk and high-risk patients. Over half of the institutions (52.2% and 68.0% in low-risk and high-risk patients, respectively) also indicated prolonged postoperative use of topical antibiotics (> 2 weeks). Not all surveyed institutions had established policies/protocols for perioperative antibiotic use in cataract surgery, endophthalmitis surveillance, and/or a monitoring program for emerging antimicrobial resistance.

**Conclusion:**

There are variations in antimicrobial prophylaxis approaches to preoperative, intra-operative and postoperative regimens in cataract surgery in Asia. More evidence-based research is needed to support the development of detailed guidelines for perioperative antibiotic prophylaxis to reduce postoperative infections.

## Introduction

Postoperative infections in ocular surgery are relatively rare—in Asia, the reported rates for endophthalmitis vary across countries and centers: 0.076–0.11% in China [1, 2]; 0.02–0.09% in India (culture-positive endophthalmitis) [3]; 0.025% in Japan [4]; and 0.063% in Korea [5]. However, cataract surgery is so widely performed that the absolute number of patients with this complication can pose a significant public health concern due to the associated visual morbidity and medical cost [6]. Cataract surgery is already the most common ocular surgery performed worldwide, and the number of cataract surgeries will continue to increase with the aging of the world population. Given the severe impact of postoperative endophthalmitis, effective infection prevention strategies are imperative. Perioperative prophylaxis for infection prevention in ocular surgery involves a variety of measures such as preoperative antisepsis, intracameral antibiotics, and topical antibiotics before and after surgery. Several evidence-based interventions, such as the use of povidone iodine on the eyelids and conjunctival sac immediately before surgery, and intracameral antibiotics (e.g., cefuroxime) at the conclusion of surgery, have demonstrated a clear benefit in reducing the rate of postoperative endophthalmitis [7]. However, there is no global consensus on the optimal strategy for perioperative prophylaxis for endophthalmitis [8].

Given the importance of effective infection prophylaxis strategies, there is a need to understand practice variations to advocate for best practices in infection prevention, and ultimately improve patient outcomes post-surgery. The aim of the Asian study on Perioperative Antibiotic prophylaXis for infection prevention (APAX) is to determine current institutional practice patterns for the use of perioperative antibiotics and other measures to prevent infection after cataract surgery in Asia.

## Methods

This study is a collaboration led by the Asia Cornea Society (ACS), supported by the Asia–Pacific Association of Cataract and Refractive Surgeons (APACRS) and involved anterior segment key opinion leaders from several Asian countries. Institutions participating in this study were selected based on: (1) their national importance, (2) local peer recognition, and (3) influence on national or regional clinical practice patterns. The ACS and APACRS were involved in the selection process of the study institutions. Participating institutions included Eye Center, 2nd Affiliated Hospital of Medical School, Zhejiang University; and Eye and ENT Hospital of Fudan University, Shanghai (China); Prince of Wales Hospital and Alice Ho Miu Ling Nethersole Hospital (Hong Kong); Aravind Eye Care System; Dr Rajendra Prasad Centre for Ophthalmic Sciences; All India institute of Medical Sciences, New Delhi, and L V Prasad Eye Institute (India); Indonesian National Eye Centre, Cicendo Eye Hospital, Bandung; JEC Eye Hospital and Clinics; and Cipto Mangunkusumo Hospital, Jakarta (Indonesia); Osaka University Hospital; and Tokyo Dental College (Japan); International Islamic University Malaysia (Malaysia); Aga Khan University Hospital (Pakistan); St. Luke’s Medical Center; and Philippine General Hospital, University of the Philippine Manila (Philippines); Singapore National Eye Centre (Singapore); Seoul National University Bundang Hospital; and Severance Hospital, Yonsei University College of Medicine (South Korea); Far Eastern Memorial Hospital, Ban Chiao District, New Taipei City; National Taiwan University Hospital; Taipei Veterans General Hospital; and Chang Gung Memorial Hospital (Taiwan); King Chulalongkorn Memorial Hospital; and Siriraj Hospital, Mahidol University (Thailand); Vietnam National Eye Hospital; and Da Nang Eye Hospital (Vietnam). A leading key opinion leader from each selected institution, typically a senior cataract or anterior segment surgeon, then completed the survey on behalf of the institution based on their knowledge of their institution practice. All respondents involved in this study formed the APAX Consortium (refer to Acknowledgements).

The survey covered three main areas in infection prophylaxis: (1) *preoperative* (before draping of the patient); (2) *intra-operative* (from applying the surgical drapes to final closure of the case and removal of drapes); and 3) *postoperative* (after the completion of the surgery; including administration of drugs in the postoperative area). The survey also divided patients in two broad categories: (1) *high-risk patients*, referring to those with any systemic or ocular condition that may require a more aggressive prophylaxis regime, including but not limited to: elderly patients (> 80 years); immunosuppressed patients or those under immunosuppressive therapies; patients with ocular surface disease; patients with prior ocular surgeries; and potentially more complex or higher risk cataract surgery (e.g., subluxated cataract, traumatic cataract, one-eyed patients, etc.,); (2) *low-risk patients*, referring to those who require routine cataract surgery and do not fulfill the criteria for ‘high risk’ as described above.

A link to the online survey (via Survey Monkey) and a softcopy version of the survey was sent via email to all members of the APAX consortium. Responses were collected over a period of 2 months (August–September 2021). The data were collated and anonymized during analysis. Data were analyzed in Microsoft Excel (version 2212), and data were classified as categorical univariate and multivariate. A test of normality was assessed using Shapiro–Wilk test. Categorical univariate data were tested for significance using *N* − 1 Chi-square test of proportions (MedCalc Software Ltd. Version 20.115), assuming a significance level of 0.05.

## Results

All 26 members of the consortium representing their respective institutions completed the survey and were included in the analysis (Table 1). Approximately half of the institutions (*n* = 12) of the institutions perform more than 5000 cataract surgery annually.

**Table 1:** Demographics of survey respondents by location and institutional surgical volume

Location	*N*
China	2
Hong Kong	1
India	3
Indonesia	3
Japan	2
Malaysia	1
Pakistan	1
Philippines	2
Singapore	1
South Korea	2
Taiwan	4
Thailand	2
Vietnam	2
Total	26

Institution annual cataract volume	*N*
< 3000 cases	9
Between 3000 and 5000 cases	5
Between 5000 and 10,000 cases^a^	4
> 10,000 cases^b^	8

^a^Eye Center, 2nd Affiliated Hospital of Medical School, Zhejiang University, China; Chang Gung Memorial Hospital, Taiwan; Taipei Veterans General Hospital; Taiwan; Siriraj Hospital, Mahidol University, Thailand

^b^Eye and ENT Hospital of Fudan University, Shanghai, China; All India Institute of Medical Sciences, New Delhi, India; Aravind Eye Care System, India; LV Prasad Eye Institute, India; JEC Eye Hospitals and Clinics, Jakarta, Indonesia; Singapore National Eye Centre, Singapore; Da Nang Eye Hospital, Vietnam; Vietnam National Eye Hospital, Vietnam

### Preoperative prophylaxis

Respondents were surveyed on the prophylactic use of preoperative topical antibiotics (Table 2). Approximately 2/3 of institutions prescribed preoperative antibiotics—61.5% (*n* = 16) in low-risk and 69.2% (*n* = 18) in high-risk cases (*p* value 0.56). Commonly used topical antibiotics included levofloxacin (56.3% [*n* = 9] in low-risk cases; 61.1% [*n* = 11] in high-risk cases), moxifloxacin (50.0% [*n* = 8] in low-risk cases; 61.1% [*n* = 11] in high-risk cases), gatifloxacin (31.3% [*n* = 5] in low-risk cases; 27.8% [*n* = 5] in high-risk cases) and tobramycin (31.3% [*n* = 5] in low-risk cases; 22.2% [*n* = 4] in high-risk cases; *p* value insignificant). Within each institution, the choice of antibiotics appeared to be similar between high- and low-risk patients, but patients at higher risk tended to be on topical antibiotics for longer periods of time prior to surgery; 37.6% (*n* = 6) versus 55.5% (*n* = 10) of surveyed institutions prescribed two or more days of preoperative antibiotics in low-risk and high-risk patients, respectively (Table 2).

**Table 2:** Preoperative use of topical antibiotics

	Low-risk patients	High-risk patients
Routine use of topical antibiotics, % (*N*)		
Yes	53.8 (14)	53.8 (14)
Sometimes	7.7 (2)	15.4 (4)
No	38.5 (10)	30.8 (8)

	Low-risk patients^a^	High-risk patients^b^
Commonly used topical antibiotics, % (*N*)		
0.5% Levofloxacin	43.8 (7)	44.4 (8)
1.5% Levofloxacin	12.5 (2)	16.7 (3)
0.5% Moxifloxacin	50.0 (8)	61.1 (11)
0.3% Gatifloxacin	31.3 (5)	27.8 (5)
0.3% Ofloxacin	12.5 (2)	11.1 (2)
0.3% Ciprofloxacin	6.3 (1)	5.6 (1)
0.3% Tobramycin	31.3 (5)	22.2 (4)
Others	6.3 (1)^c^	16.7 (3)^d^

	Low-risk patients^a^	High-risk patients^b^
Preoperative initiation, % (*N*)		
On the day of surgery	18.8 (3)	11.1 (2)
1d pre-op	43.8 (7)	33.3 (6)
2–3d pre-op	31.3 (5)	44.4 (8)
> 3d pre-op	6.3 (1)	11.1 (2)

^a^Total of 16 respondents

^b^Total of 18 respondents

^c^Others included 0.5% chloramphenicol (*n* = 1)

^d^Others included 0.5% chloramphenicol (*n* = 1), 3% gentamycin (*n* = 1) and 4% sulfamethoxazole (*n* = 1)

All respondents indicated the use of povidone iodine on the skin and in the conjunctival sac prior to surgery in all patients, with the exception of one respondent who noted that conjunctival instillation of povidone iodine was not performed in low-risk patients in their institution (Table 3). The majority of institutions (*n* = 25) applied either 5% or 10% povidone iodine on the skin (in both low-risk and high-risk settings) with one institution using 3.5% povidone iodine for the skin. Povidone iodine 5% was used for conjunctival sac instillation in both low-risk (76%; *n* = 19) and high-risk setting (76.9%; *n* = 20). However, about 20% of respondents (*n* = 5 in both low-risk and high-risk settings) indicated the use of even lower concentrations of povidone iodine, ranging from 0.25 to 3.5%, for conjunctival sac instillation in their institutions. The typical contact time of the iodine on the skin or in the conjunctival sac was 1–3 min; this was similar for both low-risk and high-risk patients (Table 3). About 30% of respondents applied povidone iodine for much longer times, up to 5 min on the skin (*n* = 8 in both low-risk and high-risk patients), and up to 10 min in conjunctival sac (*n* = 8 in both low-risk and high-risk patients). Less than 10% of respondents applied the povidone iodine on the skin (*n* = 2 in low-risk patients; *n* = 1 in high-risk patients) and in the conjunctival sac (*n* = 2 in both low-risk and high-risk patients) for less than 1 min.

**Table 3:** Preoperative use of povidone iodine

Concentration used	For skin application, % (*N*)		For conjunctival sac instillation, % (*N*)	
	Low-risk patients	High-risk patients	Low-risk patients^a^	High-risk patients
5%	30.8 (8)	30.8 (8)	76 (19)	76.9 (20)
10%	65.4 (17)	65.4 (17)	4 (1)	3.8 (1)
Others	3.8 (1)^b^	3.8 (1)^b^	20 (5)^c^	19.2 (5)^c^

Contact time	For skin application, % (*N*)		For conjunctival sac instillation, % (*N*)	
	Low-risk patients	High-risk patients	Low-risk patients^a^	High-risk patients
< 1 min	7.7 (2)	3.8 (1)	8 (2)	7.7 (2)
1–3 min	61.5 (16)	65.4 (17)	60 (15)	61.5 (16)
> 3 min	30.8 (8)	30.8 (8)	32(8)	30.8 (8)

^a^Total of 25 respondents; one respondent does not instill preoperative povidone iodine in the conjunctival sac in low-risk setting

^b^Others included 3.5% povidone iodine

^c^Others included 0.25%, 0.33%, 0.7%, 2.5% and 3.5% povidone iodine

### Intra-operative prophylaxis

According to 73.1% of respondents (*n* = 19 in both low-risk and high-risk settings), intra-operative antibiotics were used routinely and the choice of antibiotics appeared to be similar between low- and high-risk patients (*p* value 0.001) (Table 4). Preferred routes of antibiotic administration included topical drops (70% [*n* = 14] in low-risk patients; 66.7% [*n* = 14] in high-risk patients) and intracameral injections (60% [*n* = 12] in low-risk patients; 66.7% [*n* = 14] in high-risk patients) at the conclusion of surgery (*p* value 0.45). The most commonly used topical antibiotics were levofloxacin, moxifloxacin and gentamicin, while moxifloxacin, cefuroxime and ceftazidime were the most commonly used intracameral antibiotics in both low-risk and high-risk patients. Respondents who did not routinely use intra-operative antibiotics confirmed that no other adjunctive agents were used in their institution.

**Table 4:** Intra-operative use of antibiotics and the preferred route of administration

	Low-risk patients	High-risk patients
Routine use of antibiotics, % (*N*)		
Yes	73.1 (19)	73.1 (19)
Sometimes	3.8 (1)	7.7 (2)
No	23.1 (6)	19.2 (5)

	Low-risk patients^a^	High-risk patients^b^
Preferred route of administration and commonly used antibiotics, % (*N*)		
**Topical, at the end of surgery**	**70.0 (14)**	**66.7 (14)**
Levofloxacin (0.5% and 1.5%)	35.0 (7)	33.3 (7)
Moxifloxacin	30.0 (6)	33.3 (7)
Gentamicin	10.0 (2)	9.5 (2)
Cefazolin	5.0 (1)	4.7 (1)
Maxitrol	5.0 (1)	4.7 (1)
**Intracameral, at the end of surgery**	**60.0 (12)**	**66.7 (14)**
Levofloxacin (0.5%)	15.0 (3)	14.3 (3)
Moxifloxacin	35.0 (7)	33.3 (7)
Cefuroxime or ceftazidime	25.0 (5)	23.8 (5)
Vancomycin	–	4.7 (1)
**Irrigating solution during surgery**	**15.0 (3)**	**19.0 (4)**
Cefazolin	5.0 (1)	4.7 (1)
Vancomycin	5.0 (1)	9.5 (2)
Dibekacin	5.0 (1)	4.7 (1)
**Subconjunctival, at the end of surgery**	**5.0 (1)**	**9.5 (2)**
Gentamicin	5.0 (1)	9.5 (2)
Cefazolin	–	4.7 (1)

^a^Total of 20 respondents; each respondent could provide up to 3 commonly used antibiotics

^b^Total of 21 respondents; each respondent could provide up to 3 commonly used antibiotics

### Postoperative prophylaxis

In general, the prescription pattern of postoperative topical antibiotics was similar between low-risk and high-risk patients (Table 5). Antibiotics were either given as a standalone eye drop or as a component of a fixed-dose combination (FDC) of an antibiotic and steroid in a single eye drop. Among respondents who use standalone antibiotic eye drops, 3 respondents (in low-risk cases) and 4 respondents (in high-risk cases) indicated that a second FDC antibiotic-steroid eye drop was also prescribed concurrently (data not shown). Most institutions did not report the routine use of systemic antibiotics in low-risk patients, but these appear to be considered in high-risk patients (38.5% [*n* = 10] vs 3.8% [*n* = 1] in low-risk patients).

**Table 5:** Use of postoperative topical and systemic antibiotics in low-risk and high-risk settings

	Low-risk patients, % (*N*)			High-risk patients, % (*N*)		
	Topical, standalone antibiotic	Topical, fixed-dose combination antibiotic and steroid	Systemic antibiotic	Topical, standalone antibiotic	Topical, fixed-dose combination antibiotic and steroid	Systemic antibiotic
Yes/always	80.8 (21)	30.8 (8)	3.8 (1)	84.6 (22)	26.9 (7)	7.7 (2)
Sometimes	7.7 (2)	19.2 (5)	3.8 (1)	11.5 (3)	15.4 (4)	38.5 (10)
No/never	11.5 (3)	50.0 (13)	92.3 (24)	3.8 (1)	57.7 (15)	53.8 (14)

Commonly used topical, standalone antibiotics include levofloxacin, moxifloxacin and gatifloxacin (Table 6). Topical, standalone antibiotics are typically prescribed for more than 2 weeks by 52.2% [*n* = 12] and 68.0% [*n* = 17] of respondents in low-risk and high-risk cases, respectively. In terms of FDC eye drops, the tobramycin/dexamethasone combination was the most commonly reported, followed by a combination of neomycin sulfate/polymyxin B/dexamethasone (Table 7). The majority of the institutions routinely prescribe topical FDC for more than 2 weeks in low-risk (53.8%; *n* = 7) and high-risk cases (63.6%; *n* = 7).

**Table 6:** Commonly used topical, standalone antibiotic and its duration of use after surgery

	Low-risk patients^a^	High-risk patients^b^
Commonly used topical antibiotics, % (*N*)		
0.5% Levofloxacin	60.9 (14)	56.0 (14)
1.5% Levofloxacin	21.7 (5)	20.0 (5)
0.5% Moxifloxacin	56.5 (13)	60.0 (15)
0.3%/0.5% Gatifloxacin	26.1 (6)	24.0 (6)
0.3% Ofloxacin	13.0 (3)	16.0 (4)
0.3% Norfloxacin	–	4.0 (1)
0.3% Tobramycin	13.0 (3)	8.0 (2)
Others^c^	17.4 (4)	20 (5)
Postoperative duration of use, % (*N*)		
1 week or less	8.7 (2)	–
1–2 weeks	34.78 (8)	28.0 (7)
> 2 weeks	52.17 (12)	68.0 (17)
Others^d^	4.35 (1)	4.0 (1)

^a^Total of 23 respondents

^b^Total of 25 respondents

^c^Others included 0.3% gentamycin, 0.5% chloramphenicol and 4% sulfamethoxazole

^d^Depending upon case

**Table 7:** Commonly used topical, fixed-dose combination antibiotic-steroid eyedrop and its duration of use after surgery

	Low-risk patients^a^	High-risk patients^b^
Commonly used topical fixed-dose antibiotic-steroid % (*N*)		
Tobramycin/dexamethasone	53.8 (7)	54.5 (6)
Neomycin sulfate/polymyxin B/dexamethasone	30.8 (4)	27.3 (3)
Moxifloxacin/dexamethasone	–	9.1 (1)
Others	23.1 (3)^c^	18.2 (2)^d^
Postoperative duration of use, % (*N*)		
1 week or less	7.7 (1)	9.1 (1)
1–2 weeks	38.5 (5)	27.3 (3)
> 2 weeks	53.8 (7)	63.6 (7)

^a^Total of 13 respondents

^b^Total of 11 respondents

^c^Others included prednisolone acetate/ofloxacin, betamethasone/fradiomycin sulfate, dexamethasone/chloramphenicol

^d^Others included betamethasone/fradiomycin sulfate, dexamethasone/chloramphenicol

### Institutional protocols for endophthalmitis prophylaxis and surveillance

**Fig. 1:**
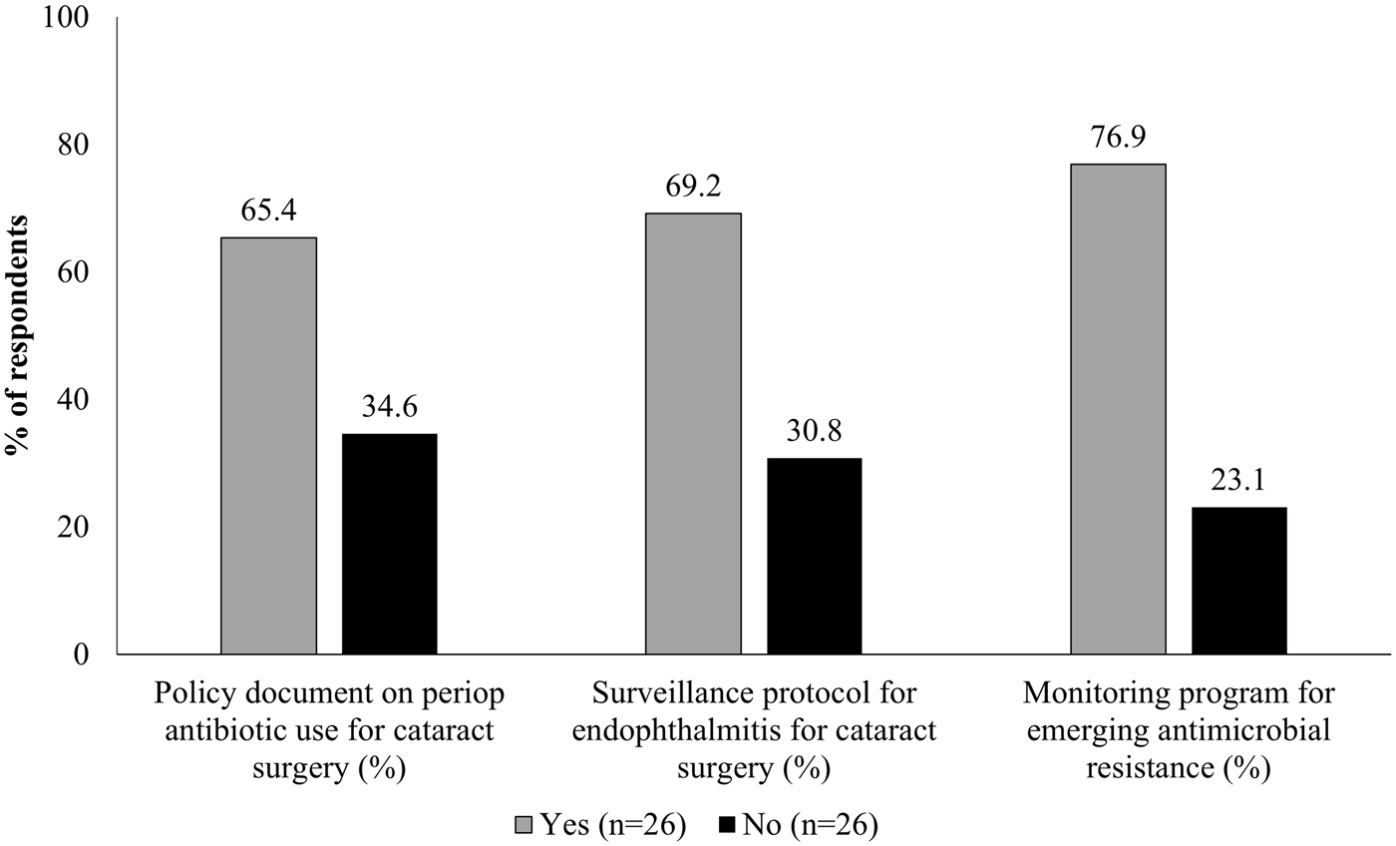
Availability of institutional protocols for antibiotic use and endophthalmitis surveillance

Respondents reported established institutional protocols in the following areas: for perioperative antibiotic use in cataract surgery (65.4% [*n* = 17]; *p* value 0.03); endophthalmitis surveillance (69.2% [*n* = 18]; *p* value 0.006); and/or monitoring program for emerging antimicrobial resistance (76.9% [*n* = 20]; *p* value 0.0016) (Fig. 1). The protocols for perioperative antibiotic use in cataract surgery are typically reviewed and revised once every 1–2 years (52.9%; *n* = 9) or every 3 years (17.6%; *n* = 3; data not shown).

## Discussion

The APAX study provides important insights into the current practice patterns for antimicrobial prophylaxis for cataract surgery across Asia and reveals variations in the approach to preoperative, intra-operative and postoperative regimes. These should be compared to current evidence-based and best-protocols, with the proviso that in many instances, empirical and pragmatic approaches need to be applied as clear consensus may be lacking.

While various preferred practice patterns on adult cataract surgery exist, such as the American Academy of Ophthalmology (AAO) and the APACRS guidelines [7, 9], the ground realities on such practices may vary. A survey over a wide and populous geographic region such as Asia brings forth the variabilities and lack of consensus regarding practice patterns. Such a survey will inform any deviations from accepted recommendations for safe and preferred perioperative practices supported by a wide body of literature [10]. Furthermore, regular surveys over a cross section of practicing ophthalmologists can highlight how well recommended guidelines have been implemented. Similar surveys do exist, such as that by Maharana et al. [3]—as such, conducting surveys in comparable cohorts helps assess the changes in practice patterns over time.

Preoperative antisepsis with topical povidone iodine has been studied extensively and has proven to be the most effective method for preventing postoperative endophthalmitis [11]. The use of povidone iodine is a standard of care in ophthalmic surgery [12], and this is reflected in our study. The current AAO preferred practice guidelines note the efficacy of preoperative instillation of 5% povidone iodine in the conjunctival sac in reducing bacterial load and the incidence of postoperative infection [7]. The European Society of Cataract and Refractive Surgeons (ESCRS) guidelines recommend the application of 5–10% povidone iodine to the cornea, conjunctival sac and periocular skin for at least 3 min before surgery [12]. In our study, the majority used 10% povidone iodine for the periocular skin application (65%) and 5% povidone iodine for conjunctival sac instillation (76%). About 20% of respondents used lower concentrations (0.25–3.5%) of povidone iodine in the conjunctival sac. Some studies have shown that dilute concentrations of povidone iodine (0.05–1%) have better bactericidal activity, owing to the greater availability of free iodine, and could reduce cornea toxicity [13, 14]. These findings may provide some rationale for the use of lower concentration (< 5%) of povidone iodine, although there are currently no guidelines recommending this practice for conjunctival sac instillation. The minimum contact time of povidone iodine recommended by the ESCRS guidelines is 3 min [12], and this has been demonstrated to be essential in reducing organism from the lid and conjunctival flora [15]. However, about 50% of respondents in our study indicated contact time of povidone iodine that is less than the recommended minimum of 3 min, with a small percentage applying the iodine for less than 1 min—this foreshortened contact times, probably relating to practical issues, should be reviewed.

Preoperative topical antibiotic prophylaxis is intended to decrease the bacterial load on the ocular surface, thereby reducing the risk of bacterial contamination during surgery. However, there is a lack of good evidence that this reduces the incidence of postoperative endophthalmitis, especially in the presence of povidone iodine use [13, 16]. The 2013 ESCRS guidelines and the 2021 AAO preferred practice guide concluded that no clear benefit has been established for preoperative antibiotics, and their use may be unnecessary and may increase the potential risk of bacterial resistance [7, 12]. Indeed, there is wide variability in the use of preoperative topical antibiotic across different countries: 33.2% in Australia/New Zealand, 85% in the USA, 100% in China [8]; 90% in India [3]; and 99% in Japan [17]. Furthermore, preoperative antibiotic use has reportedly declined in many European countries in recent years [11], and the recent 2021 ASCRS survey also noted a drop in the use of preoperative antibiotics (73% in 2021 vs 85% in 2014 survey) [18]. The decline of preoperative topical antibiotic use in these countries may be due to the increasing use of intracameral antibiotics in cataract surgery [18]. Our findings of approximately 2/3 of institutions in this study adopting the use of preoperative topical antibiotics thus present an opportunity for educating ophthalmologists in Asia, especially in view of higher prevalence of antimicrobial resistance in many countries in the region.

With respect to the duration of use of preoperative topical antibiotics, some studies suggest that instillation of topical antibiotics on the day of surgery or 1 day prior to surgery may be sufficient to reduce conjunctival flora [19–21]. However, another study found a 3-day prophylactic regimen to be more effective than on-the-day 1-h regimen [22]. He et al. also reported increased antibiotic resistance with a 1-day preoperative prophylaxis regimen versus a 3-day regimen [23]. A survey of Japanese surgeons reported that 83% initiated preoperative antibiotic eye drops 2–5 days before surgery [17]. In India, 44.5% use preoperative topical antibiotics 1-day before surgery and 44.2% use it 3-days prior to surgery [3]. Among the ASCRS members, 30% use preoperative topical antibiotics 1-day before, 47% 3-day before and 23% on the day of the surgery [18]. Likewise in our study, ~ 75% prescribe topical antibiotics 1–3 days prior to surgery, and there is a tendency for a longer duration of use (≥ 2 days before surgery) in high-risk patients.

With regard to intra-operative use of antibiotics, there is growing evidence demonstrating the benefit of intracameral antibiotics in reducing the rate of postoperative endophthalmitis [16, 24–29]. The 2007 ESCRS study also found that intra-operative topical antibiotics provide incremental benefit to intracameral cefuroxime, albeit not statistically significant [24]. Another study however reported that there was no difference in efficacy between intracameral antibiotics alone versus intracameral plus topical antibiotics [16], suggesting that intra-operative topical antibiotics may be unnecessary when intracameral antibiotics are used. In our study, there is a similar preference for topical and intracameral use of antibiotics, suggesting that one may be used as an alternative or adjunctive to the other. Almost two-thirds of institutions in our study used intracameral antibiotics during cataract surgery, which is commensurate with the wide range reported in the literature where usage ranges from 7% in Japan to 50% in the US [8]. Only 40% of the surveyed members of the All India Ophthalmological Society used intracameral antibiotics after cataract surgery; of these, 46.2% use it for high-risk cases only while 36.6% use it in all cases [3]. Commonly cited barrier to the greater usage of intracameral antibiotics is the lack of a commercial preparation approved for use in the country [30], resulting in off-label use of other agents. Agents that are not prepared specifically for intracameral application may contain ingredients that may not be optimal for such a purpose. Furthermore, dilution errors may result in ocular toxicity [30].

Postoperative topical antibiotics are generally more frequently used (> 95%) across different countries [8]. The ESCRS guidelines recommend postoperative antisepsis at the discretion of the surgeon based on the postoperative environment, occurrence of surgical complications and presence of other patient- or procedure-related risk factors [12]. About 88–95% of respondents in our study use postoperative topical antibiotics, and prior studies have reported similar rates in Japan (100%) [17] and in India (94.4%) [3]. In general, antibiotics should be used appropriately by limiting its duration of use and selecting one that is most effective to the pathogens commonly known to cause the disease; however, there is thus far no preferred or standardized regimen for postoperative use of topical antibiotics. The majority of respondents (~ 70%) in the recent ASCRS survey discontinued postoperative topical antibiotics within 1 week, while the remaining continued them for several weeks [18]. In our study, the majority of institutions prescribed postoperative topical antibiotics for 2–4 weeks after surgery (between 52.2 and 68.0%).

The availability of vitreous samples for microbial profiling could guide in selecting the appropriate postoperative topical antibiotics. The most common organisms involved in post-cataract surgery endophthalmitis are Coagulase-negative staphylococcus (CoNS), *Staphylococcus aureus*, *Staphylococcus epidermidis*, and gram-positive streptococcus [31, 32]. Fluoroquinolones tend to be preferred due to their relatively broad spectrum against most Gram-positive and -negative organisms, ability to penetrate the corneal epithelium, and commercial availability [12, 33]. This preference is seen in the recent ASCRS survey [18] and also in our study. However, increasing rates of resistance to broad-spectrum antibiotics such as methicillin, cephalosporin, fluroquinolones and aminoglycosides have been reported worldwide. The organisms that have shown resistance include *Staphylococcus aureus*, CoNS, *Streptococcus spp*., *Pseudomonas aeruginosa* and *Moraxella spp*. [34–39]. As such, the postoperative use of prophylactic antibiotics in the setting of a routine cataract surgery for more than 2 weeks should be reconsidered in light of evidence that repeated or prolonged postoperative use of antibiotics increases antibiotic resistance of the microbial flora on the ocular surface [40–42].

The present study has some limitations. Firstly, the responsibility of accurate data collection for the survey was placed on individual senior key opinion leaders who, one would assume, would have to try and generalize their institutions’ practice patterns. This obviously could be challenging if there was limited consensus and significant practice variations among surgeons in individual institutions. This was partly mitigated by the presence of standard clinical protocols which were almost always in place in the perioperative processes of the institutions. Secondly, clearly just 26 centers would not necessarily be considered to be wholly representative for these Asian countries, and may not represent individual practice patterns of ophthalmologists working in smaller settings. Again, this is mitigated to some extent by the fact that the tertiary level institutions selected were generally larger or prominent, national, academic teaching institutions, and one would assume that these centers would generally influence clinical local practices by virtue of their training of the local residents.

Taken together, our survey highlights the variations in infection prophylaxis practice patterns in cataract surgery in Asia and consequently the need for more evidence-based research which could subsequently lead to the development of detailed guidelines for perioperative antibiotic prophylaxis to reduce the incidence of postoperative infection. National and regional societies could consider adopting, or at least, adapting such protocols and recommendations, especially in view of the increasing concerns over antimicrobial resistance.
